# The ad-libitum taste test as measure of momentary alcohol use in the laboratory: an investigation of construct validity and confounding factors

**DOI:** 10.1007/s00213-023-06518-0

**Published:** 2023-12-23

**Authors:** Sebastian Trautmann, Anja Kräplin, Markus Muehlhan, Fée Ona Fuchs, Beate Loesch, Charlotte Wittgens

**Affiliations:** 1 Department of Psychology, Faculty of Human Science, Medical School Hamburg, Am Kaiserkai 1, 20457 Hamburg, Germany; 2 Institute of Clinical Psychology and Psychotherapy, Medical School Hamburg, ICPP, Am Kaiserkai 1, 20457 Hamburg, Germany; 3 Work Group Addictive Behaviors, Risk Analysis and Risk Management, Faculty of Psychology, Technische University Dresden, Dresden, Germany; 4 ICAN Institute for Cognitive and Affective Neuroscience, Medical School Hamburg, Am Kaiserkai 1, 20457 Hamburg, Germany

**Keywords:** Ad-libitum taste test, Alcohol use, Laboratory, Validation

## Abstract

**Rationale:**

The ad-libitum taste test is a widely used covert measure of motivation to consume alcohol in the laboratory. However, studies on its construct validity and potential confounding factors are scarce.

**Objectives:**

This study aimed to evaluate the construct validity of the ad-libitum taste test by examining the association of ad-libitum alcohol consumption with typical alcohol use and craving, and investigating potential moderation by trait anxiety, depressiveness, current mood, and drinking motives.

**Methods:**

A sample of 264 young male individuals were offered two 0.33 l glasses of beer. Participants were instructed to rate the characteristics of each drink, while the percentage of beverages containing alcohol consumed was assessed. Associations of ad-libitum consumption with typical alcohol use and craving were assessed using non-parametric and piecewise regressions. Moreover, moderator analysis with trait anxiety, depressiveness, current mood, and drinking motives was carried out.

**Results:**

Ad-libitum alcohol consumption was associated with typical alcohol use and alcohol craving. However, these associations decreased at high consumption levels. Associations between ad-libitum consumption, typical alcohol use, and craving were stable across several conditions, except that the association between ad-libitum consumption and craving increased with higher social, conformity, and coping drinking motives.

**Conclusions:**

The ad-libitum taste test appears to be a valid measure of the motivation to drink alcohol in laboratory studies in young male adults, although this validity might be compromised at high levels of ad-libitum consumption. Consideration of these factors can contribute to further refining the ad-libitum taste test as a valuable tool for assessing motivation to consume alcohol in laboratory studies.

## Introduction

Excessive alcohol consumption contributes to various illnesses and injuries, leading to premature morbidity and mortality (Rehm et al. 2010; Lim et al. 2012). Men consume about three times as much alcohol as women and suffer from more alcohol-related harms than women (White 2020). In order to reduce the individual and societal burden of excessive alcohol use, it is crucial to identify causal factors, moderating variables, and underlying mechanisms contributing to the development of excessive alcohol use and alcohol use disorders. Apart from well-designed longitudinal studies, which are costly and time-consuming to conduct, research on the causal mechanism of alcohol use relies primarily on experimental paradigms and carefully controlled laboratory designs (Bujarski and Ray 2016).

The ad-libitum taste test is a widely used method that provides an unobtrusive and indirect measure of participants’ motivation to drink alcohol. Participants are given access to a range of beverages containing alcohol and are asked to rate these beverages on a series of adjectives. The taste ratings are intended to conceal the genuine purpose of the taste test, which is to assess the amount of alcohol consumed during the test session (Jones et al. 2016). The taste test has been used to investigate a number of potential influences on alcohol consumption, including alcohol cues (Colby et al. 2004; Jones et al. 2013; Van Dyke and Fillmore 2015), impulse control (Jones et al. 2011; Christiansen et al. 2012), stress (Thomas et al. 2011; Bacon and Thomas 2013; McGrath et al. 2016), coping motives (Thomas et al. 2014), and social influences (Quigley and Collins 1999), and it has been used to establish initial proof of concept for novel interventions (Field and Eastwood 2005; Bowley et al. 2013; McNeill et al. 2022).

Although the ad-libitum taste test has been employed in numerous studies, its robustness and construct validity have been rarely investigated so far. In two studies using an ad-libitum alcohol consumption paradigm without the objective of validating taste test, there was no or only partial evidence (for one out of four measures from the taste test) that ad-libitum alcohol use could be predicted by typical alcohol use (Leeman et al. 2009, 2013). The most comprehensive study to validate the ad-libitum taste test (Jones et al. 2016) in young male and female adults found that ad-libitum consumption was associated with both typical alcohol consumption and alcohol craving, while it was not related to time of day as a potential confounding factor, providing initial support for the construct validity of the ad-libitum taste test. With the present study, we aimed to build on the work of Jones et al. (2016). First, we aimed to replicate in a sample of male young adults the finding that ad-libitum consumption is associated with both typical alcohol consumption and alcohol craving. Our main objective was then to assess the robustness of these associations by investigating whether they depend on different variables including age, anxiety, depressiveness, mood, and drinking motives, which are likely to vary in different samples and which have all been related to alcohol-related measures in laboratory studies (Field and Quigley 2009; Wiers et al. 2015; Bresin et al. 2018).

## Materials and methods

### Design

To investigate the proposed research questions, we used data from a research project investigating the underlying mechanisms in the association between stress and alcohol use in male healthy individuals (Wittgens et al. 2022). This study consisted of an online screening, a comprehensive questionnaire assessment, a random assignment to either a stress induction or a control condition, behavioral paradigms, and the ad-libitum taste test as the main study outcome. The data relevant to this paper stem from the online screening, the questionnaire assessment, and the ad-libitum taste test which were collected between November 2018 and August 2022 in a laboratory setting in Hamburg, Germany. The study was approved by the local ethics committees (EK 522122016, MSH-2020/114).

### Participants

The original study assessed a convenience sample of *n* = 283 male participants. Individuals were included in the original study if they were between 18 and 40 years old and drank alcohol at least occasionally. Additionally, a hair length of at least 2 cm was required to obtain biological measures not relevant to this paper. Exclusion criteria included lifetime psychotic symptoms, lifetime alcohol or any other substance use disorder, current psychological or psychopharmacological interventions and acute suicidality, current psychotropic or other medication, or any severe somatic diseases. All participants for whom data were available from the ad-libitum taste test, typical alcohol use, and craving (*n* = 264) were included in this analysis. As part of the published study protocol (see Wittgens et al. 2022), an a priori Monte Carlo simulation power calculation was conducted for bivariate associations and two-way interactions. We calculated that a sample size of *n* = 400 would assure power between 0.80 and 0.95 for effect sizes between *d* = 0.3 and *d* = 0.5. Since the study was conducted during the COVID pandemic with associated lab closures (also described in the study protocol), the sample size of 400 was not reached. Thus, several interaction analyses should be interpreted with caution (also, see the “Discussion” section for a discussion of limitations).

### Study measures

#### Ad-libitum taste test

Participants were asked to take part in an ad-libitum taste test as a covert measure of alcohol consumption. The taste test took place in the psychology labs in an empty and quiet room where drinks were placed on a table in a standardized order. All participants were given two 0.33 l glasses of beer (mean = 673.3 g, SD = 12.8 g) (German brands “Holsten” and “Astra”, containing 4.8% and 4.9% alcohol per volume, respectively) and two 0.33 l glasses of different soft drinks (mean = 714.2 g, SD = 64.7 g) (apple soda). Beverages without alcohol were presented to be able to adjust the analyses for the potential effect of thirst. The shapes of the glasses were the same for all drinks. Participants were instructed to take 15 min to taste each glass and to rate the characteristics of each drink (e.g., gassy, bitter). Participants were instructed to drink as much as they needed to make accurate judgements. The dependent variable was the percentage of consumed beer out of all beer available.

### Construct validity

#### Typical alcohol consumption

Typical alcohol consumption was measured using a web-based Self-Administered Timeline Follow Back (STLFB) consisting of a calendar in which participants retrospectively record their alcohol consumption in the past 30 days (Collins et al. 2008), separately for weekdays, weekends, and special drinking occasions when participants reported drinking more than usual (Stahre et al. 2006). The average daily alcohol consumption in grams was calculated to be used as a variable in the subsequent analyses.

#### Current alcohol craving

The revised version of the Alcohol Craving Questionnaire (ACQ-R) (Raabe et al. 2005) is a self-report instrument designed to assess cravings for alcohol in individuals with alcohol use disorder. It consists of 12 items that focus on the intensity, frequency, and duration of alcohol craving. Each item is rated on a Likert scale ranging from 1 (strongly disagree) to 7 (strongly agree). The internal consistency was *α* = 0.86 in the present sample.

### Potential confounding variables

#### Current mood

Current mood was assessed with the mood subscale of the Multidimensional Mood State Questionnaire (MDBF) (Hinz et al. 2012), a self-report questionnaire designed to measure an individual’s mood state across three dimensions: energetic arousal, mood (valence), and tense arousal. The MDBF mood subscale consists of 8 items rated on a 5-point Likert scale. The internal consistency of the mood subscale was *α* = 0.92.

#### Trait anxiety and depressive symptoms

The State-Trait Anxiety Inventory (STAI) (Spielberger 1983) is a widely used psychological assessment tool designed to measure trait and state anxiety levels in individuals. The trait anxiety measure of the STAI contains 20 items, which are rated on a 4-point Likert scale ranging from “Almost Never” to “Almost Always.” These items assess how an individual typically feels or behaves in response to stressors, and higher scores indicate a higher level of trait anxiety. To assess depressive symptoms, we used the Beck Depression Inventory-II (BDI-II) (Hautzinger et al. 2006). The BDI-II consists of 21 items, with each item presenting a group of four statements reflecting different degrees of a specific depressive symptom. Participants are asked to choose the statement that best describes how they have been feeling over the past 2 weeks, including the day of assessment. Each response is scored from 0 to 3, with higher scores indicating more severe symptoms. The total score ranges from 0 to 63, with higher scores indicating greater depression severity. The internal consistencies for the trait anxiety measure of the STAI and the BDI-II were *α* = 0.90 and *α* = 0.87, respectively.

#### Drinking motives

The Drinking Motives Questionnaire-Revised (DMQ-R) (Cooper 1994) is a self-report instrument designed to assess the motives underlying alcohol consumption. It consists of four subscales referring to the following four primary motives: social, enhancement, coping, and conformity. The DMQ-R contains 20 items, with five items for each of the four motive categories. Respondents rate each item on a 5-point Likert scale, ranging from 1 (almost never/never) to 5 (almost always/always), to indicate how frequently they consume alcohol for each specific reason. The internal consistencies for the four subscales ranged between *α* = 0.60 and *α* = 0.65.

### Procedure

The main assessments were conducted on weekdays between 2 and 8 pm. First, participants were asked to provide written informed consent. Participants were deceived to allow a covert assessment of alcohol use in order to reduce demand bias. Participants were told that the study aimed to examine the influence of personality traits on the ability to detect subtle differences in the taste of different beverages. However, the entire study procedure was fully explained in the consent form and participants were debriefed about the true study purpose after the assessments. Participants’ absence from alcohol was verified by taking a breathalyzer test with any value above zero leading to the immediate end of the examination. Then, participants completed a series of questionnaires including drinking motives, trait anxiety, and depressive symptoms. Subsequently, participants either participated in the Trier Social Stress Test or placebo intervention (control condition) followed by behavioral assessments (Wittgens et al. 2022). The ad libitum taste test was the last component of the study procedure. Throughout the study, mood and craving were measured repeatedly, but only measurements immediately before the taste test were used for this analysis. After the taste test, all participants were debriefed about the true purpose of the study. Moreover, breathalyzer tests were taken repeatedly until blood alcohol concentration reached 0.0‰ for two consecutive measures. Participants willing to leave before blood alcohol concentration reached 0.0‰ had to confirm that they do not drive when leaving the laboratory. All components of the entire study program are described in detail elsewhere (Wittgens et al. 2022).

### Statistical analyses

Before data analyses, the distributions of all variables were visually examined. Since the distributions of both typical alcohol use and alcohol craving were considerably skewed, associations between ad-libitum alcohol consumption, typical alcohol use, and alcohol craving were modeled with gamma regressions and robust standard errors (Royall 1986; Malehi et al. 2015). Associations are quantified with exponentiated regression coefficients (mean ratio, MR), and the corresponding 95% confidence intervals. The adjusted explained variance was also calculated according to the following formula and is reported for all models.

$$1-\frac{\left(1-{R}^{2}\right)\left(n-1\right)}{n-k-1}$$

Since the scale of ad-libitum taste test consumption would result in very small coefficients, the scale was linearly transformed with every value divided by 10, allowing an interpretation of MR as the increase in the dependent variable with every 10% increase in ad-libitum consumption. Moreover, ad-libitum consumption of beverages not containing alcohol was added to all models to adjust for thirst.

In a second step, the shapes of these associations were examined using a kernel-weighted local polynomial regression (Fan and Gijbels 1996) to investigate whether associations were actually linear or whether models would need adjustment. Nonparametric regressions make no assumptions about the functional form for the expected value of a response variable given a regressor. In case these nonparametric regressions revealed evidence for non-linear associations, piecewise regression analyses with linear splines were conducted (Newson 2012). A piecewise regression model allows for changes in the slope, with the restriction that the function to be estimated must be continuous. Thus, the estimated model is continuous, with a structural break. The change in the slope at this structural break is then tested. Linear splines allow the estimation of the relationship between two variables as such a piecewise linear function composed of linear segments (separated at the pre-defined structural breaks) and therefore allow the examination of changes in the regression slope for associations of both typical alcohol use and craving with ad-libitum alcohol consumption in case of non-linearity.

We also investigated whether associations of ad-libitum alcohol consumption with typical alcohol use and craving varied with changing levels of participants’ age, current mood, trait anxiety, depressive symptoms, and alcohol use motives. For these moderator analyses, we also fitted gamma regression models, adding interaction terms between ad-libitum alcohol consumption and the respective variable.

Since the original study design included two main groups (stress induction, control), we also added a further interaction term to the regressions to investigate whether associations and moderations differed between these two study groups.

## Results

### Sample characteristics

The mean age of the total sample was 24.8 (SD = 4.4) years, with the majority (73.1%) being university students. The average typical alcohol use per day was 19.9 g (SD = 18.9 g). Detailed characteristics of the sample are shown in Table 1, separated by the original study groups.

**Table 1:** Demographic, psychological, and substance-related sample characteristics

	Stress (*n* = 108)	Control (*n* = 156)
	*N* (%)/mean (SD)	*N* (%)/mean (SD)
Race/ethnicity		
European	103 (95.4)	149 (95.5)
North American	2 (1.9)	0 (0.0)
Central/South American	1 (0.9)	1 (0.6)
Asian	0 (0.0)	2 (1.3)
Not responded	2 (1.9)	4 (2.6)
Age	24.3 (4.4)	25.2 (4.4)
Family status		
Single	100 (92.6%)	151 (96.8%)
Married	6 (5.6%)	3 (5.6%)
Widowed/divorced	1 (0.9%)	2 (0.9%)
Highest educational degree		
Secondary school certificate	6 (5.7%)	11 (7.1%)
Higher education entrance qualification	65 (61.3%)	78 (50.3%)
University degree	33 (31.1%)	63 (40.7%)
Other	2 (1.9%)	3 (1.9%)
Budget (monthly)		
< 500€	25 (23.2%)	28 (18.0%)
500–1000€	41 (38.0%)	56 (35.9%)
1000–1500€	19 (17.6%)	35 (22.4%)
1500–2000€	4 (3.7%)	14 (9.0%)
> 2000€	19 (17.6%)	23 (14.7%)
Past 4 weeks of typical alcohol use (STLFB)		
Alcohol use total in g/d	20.3 (17.6)	19.6 (19.8)
Alcohol use on weekdays in g/d	4.4 (5.6)	3.7 (5.3)
Alcohol use on weekend days in g/d	42.5 (43.1)	43.2 (54.0)
Number of special drinking occasions	1.3 (1.4)	1.2 (1.3)
Alcohol use per special drinking occasion in g	76.8 (87.6)	76.5 (86.3)
Risky drinking	37 (34.3%)	41 (26.3%)
Alcohol craving (ACQ-r)	31.6 (13.6)	29.1 (12.3)
Current mood (MDBF)	32.5 (5.0)	33.2 (6.0)
Trait anxiety (STAI)	35.4 (9.3)	34.6 (8.4)
Depressiveness (BDI-II)	6.0 (6.1)	5.9 (5.0)
Drinking motives (DMQ-r)		
Social	10.2 (3.2)	10.0 (3.3)
Enhancement	12.9 (3.3)	13.1 (3.5)
Coping	10.3 (3.0)	10.2 (3.1)
Conformity	8.2 (2.4)	8.0 (2.4)

Special drinking occasion—occasion with higher than usual alcohol consumption in the past 4 weeks. Risky drinking—typical alcohol use > 20 g alcohol per day

*STAI* trait score of the state-trait anxiety inventory, *BDI-II* Beck depression inventory-II, *MDBF* mood subscale of the multidimensional mood state questionnaire, *STLFB* self-administered timeline follow back, *ACQ-r* alcohol craving questionnaire revised, *DMQ-r* drinking motives questionnaire revised

### Construct validity of the ad-libitum taste test

Participants consumed on average 58.3% (SD = 28.8) of the offered beverages containing alcohol, which was less than the average percentage of beverages not containing alcohol consumed (mean = 49.2% (SD = 24.6), *t*(263) =  − 3.7, *p* < 0.001). Percentage of ad-libitum alcohol consumption was positively associated with both typical alcohol use (MR = 1.09 [1.05–1.14]) *p* < 0.001, adj. *R*^2^ = 0.068) and alcohol craving immediately before the taste test MR = 1.06 [1.04–1.08]) *p* < 0.001, adj. *R*^2^ = 0.130). These associations did not differ between the two study groups (*Χ*^2^(1) < 1.72, *p* > 0.191).

**Fig. 1:**
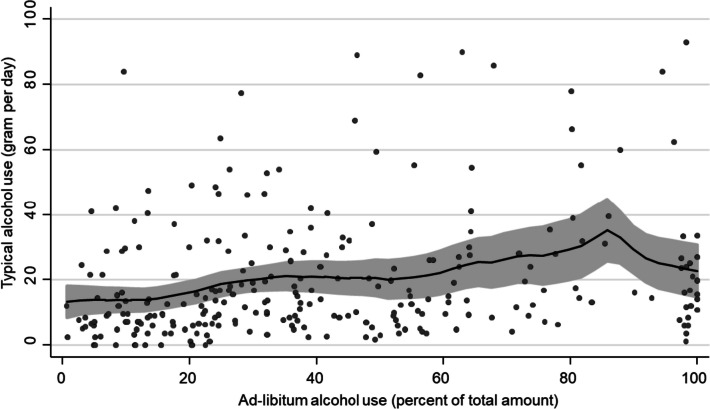
Association between ad-libitum alcohol consumption and typical alcohol use from non-parametric regression (local polynomial smoothing). Grey area indicates 95% confidence interval

Visual inspection of these associations with non-parametric regressions revealed evidence of non-linearity. For the association between ad-libitum and typical alcohol consumption, a small decrease in the association at 40% ad-libitum consumption and a steeper decrease at 85% ad-libitum alcohol consumption was observed (Fig. 1).

**Fig. 2:**
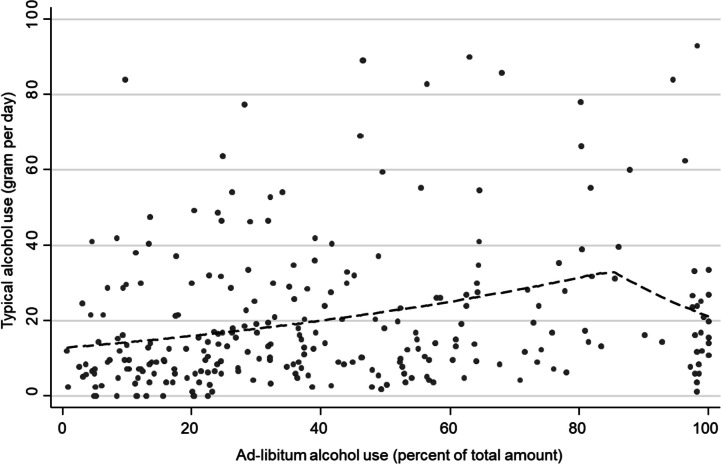
Association between ad-libitum alcohol consumption and typical alcohol use from piecewise linear regression with change point at 85% ad-libitum consumption

In piecewise regression analysis, we, therefore, tested two models for potential changes in regression slopes—one model with two change points at 40% and 85% ad-libitum consumption and one model with only one change point at 85% ad-libitum consumption. While the first model revealed no evidence of significant changes in regression slopes (slope change after 40% (MR = 0.96 [0.80–1.15] *p* = 0.652) and the slope change after 85% (MR = 0.74 [0.51–1.08] *p* = 0.120, adj. *R*^2^ = 0.082), the second model yielded a significant decrease in regression slope after 85% ad-libitum consumption (MR = 0.70 [0.50–0.97] *p* = 0.034, adj. *R*^2^ = 0.087) (Fig. 2). This change in regression slope did, again, not differ by study group (*Χ*^2^(1) = 0.24, *p* = 0.627).

**Fig. 3:**
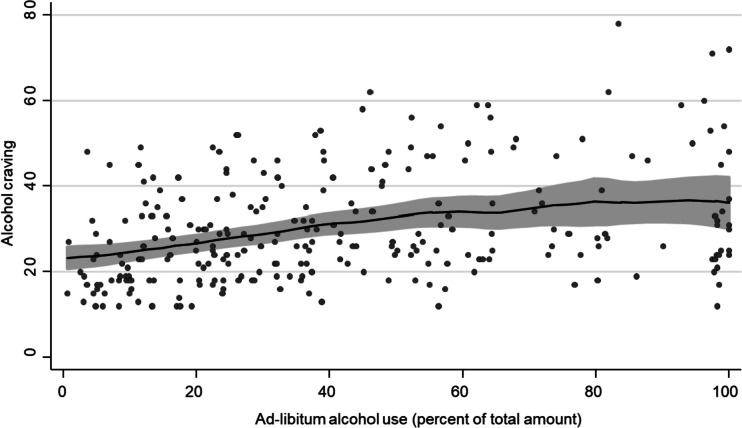
Association between ad-libitum alcohol consumption and alcohol craving from non-parametric regression (local polynomial smoothing). Grey area indicates 95% confidence interval

**Fig. 4:**
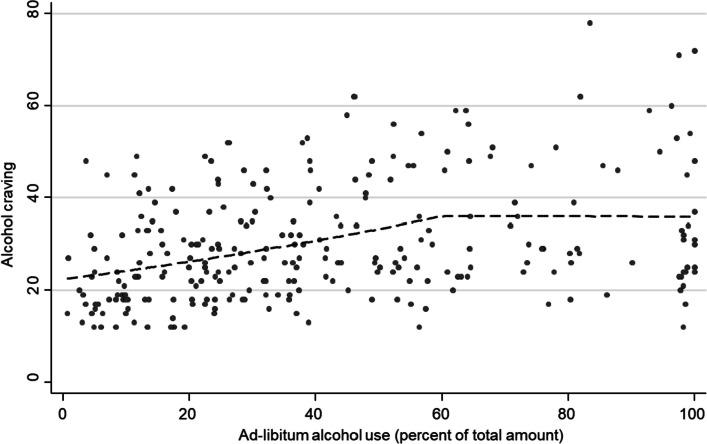
Association between ad-libitum alcohol consumption and typical alcohol use from piecewise linear regression with change point at 60% ad-libitum consumption

For the association between ad-libitum alcohol consumption and craving, the association declined more steadily with increasing levels of ad-libitum consumption, with a potential change point at 60% (Fig. 3). This could be confirmed in piecewise regression analysis, where the regression slope decreased after 60% ad-libitum consumption (MR = 0.93 [0.86–0.997] *p* = 0.041, adj. *R*^2^ = 0.141) (Fig. 4). Like in previous analyses, this change in regression slope did not differ by study group (*Χ*^2^(1) = 0.23, *p* = 0.629).

### Moderators

The complete results of the analyses of potential moderators of the association between ad-libitum alcohol consumption and typical alcohol use and craving are shown in Table 2. None of the investigated variables moderated the association between ad-libitum consumption and typical alcohol use. The association between ad-libitum consumption and craving increased with higher levels of social (MR = 1.01 [1.00–1.01] *p* = 0.017) and coping drinking motives (MR = 1.01 [1.00—1.01] p = 0.001). In the 16 moderation models, there were two differences by study group: the association between ad-libitum consumption and typical alcohol use increased with trait anxiety and decreased with better mood in the control but not in the stress group (group differences—trait anxiety, *Χ*^2^(1) = 5.62, *p* = 0.018; mood, *Χ*^2^(1) = 6.18, *p* = 0.013).

**Table 2:** Moderators of the association of ad-libitum alcohol consumption with typical alcohol use and craving

	Typical alcohol use			Craving		
	MR	95% CI	*P*	MR	95% CI	*P*
Age	1.00	0.99–1.01	0.735	0.999	0.995–1.003	0.765
Depressiveness	1.00	0.998–1.01	0.270	1.00	0.998–1.00	0.391
Trait anxiety	1.00	0.999–1.01	0.187	1.00	0.999–1.00	0.169
Mood	1.00	0.99–1.01	0.963	0.997	0.99–1.00	0.075
Drinking motive						
Social	1.01	0.995–1.02	0.255	1.01	1.00–1.01	0.017
Enhancement	1.00	0.99–1.01	0.487	1.00	0.999–1.01	0.061
Coping	1.00	0.99–1.02	0.852	1.01	1.00–1.01	0.001
Conformity	1.00	0.99–1.02	0.689	1.00	0.99–1.01	0.612

Results from gamma regression models with an interaction term of ad-libitum consumption × moderator predicting typical alcohol use and craving

*MR* exponentiated regression coefficients (mean ratio)

## Discussion

In this study, we evaluated the construct validity and potential confounding factors of the ad-libitum alcohol taste test as a covert measure of motivation to drink alcohol in laboratory studies. Our findings generally support the construct validity of the ad-libitum taste test, indicated by associations between ad-libitum alcohol consumption with the external validators’ typical alcohol use in daily life and alcohol craving immediately before the test. These findings are consistent with a previous validation study of the ad-libitum taste test (Jones et al. 2016). However, the variance of typical alcohol consumption explained by the ad-libitum consumption in the lab was rather small. In line with this observation, associations with typical alcohol use and craving were weaker at high levels of ad-libitum consumption, indicating that the test may be less valid when participants consume all or almost all of the offered beverages. There are several possible explanations for this observation. First, there is a likely ceiling effect. Although the average percentage of beverages containing alcohol consumed was about 40%, there was some clustering of participants that drank the entire amount offered. For these participants, the ad-libitum taste test may no longer differentiate between individuals, especially regarding typical alcohol use. The total amount of 660 g of beverages containing alcohol offered in this study was in the middle range compared to previous studies (Thomas et al. 2014; McGrath et al. 2016). Based on our findings, a higher amount seems to be recommended, especially in study samples with riskier drinking patterns. A second explanation could be social desirability: individuals who want to perform well on the task of evaluating the taste of the beverages may drink more with the intention of providing a more accurate assessment. This same social desirability could also be related to the underreporting of typical consumption and craving, which could at least partially explain the observations of reduced associations at high levels of ad-libitum consumption. Since gender differences in socially desirable responses are likely (Becker and Cherny 1994), the exclusively male sample limits the interpretability, and different patterns might be observed in female samples. A third explanation could be that high ad-libitum consumption reflects a strong preference for the specifically offered beverage rather than a motivation to drink alcohol per se, reducing the associations with alcohol-related measures. Asking about the pleasantness of the offered drinks (Jones et al. 2016) could help to disentangle the liking of a specific beverage from the motivation to drink alcohol.

In addition to investigating the construct validity of the ad-libitum taste test as a measure of motivation to drink alcohol, our study also explored the potential moderating effects of trait anxiety, depressiveness, current mood, and drinking motives on the associations between ad-libitum consumption and the external validators typical alcohol use and craving. Our results indicated that none of these variables moderated the association between ad-libitum consumption and typical alcohol use. This suggests that the ad-libitum taste test’s construct is robust across a range of psychosocial variables. However, we found that the relationship between ad-libitum consumption and craving was moderated by social and coping drinking motives. The association between ad-libitum alcohol use and craving increased with higher levels of these drinking motives. In other words, higher levels of reported drinking motives were related to increased craving, which in turn transferred to actual alcohol consumption in the specific ad-libitum setting. There is surprisingly little work investigating moderators of initiated drinking in case of elevated alcohol craving. One possible explanation for the moderating role of drinking motives in the association between craving and ad-libitum consumption could be the association between levels of reported drinking motives and impulsivity (Adams et al. 2012). More specifically, the impulsivity facet urgency, which refers to a tendency to engage in impulsive behavior to alleviate emotional distress, has been related to higher levels of social, coping, and conformity (but not enhancement) motives (Jones et al. 2014). Similar findings have been observed for the association between drinking motives and impaired control (Bitsoih et al. 2023). One might therefore speculate that in case of increased alcohol sensitivity (i.e., craving), individuals who report higher levels of drinking motives are more likely to engage in ad-libitum consumption through the effects of higher impulsivity and/or impaired control. This, however, remains speculative and should be subject to future investigations. Moreover, all moderating effects were rather small in magnitude, and replications, particularly in more diverse samples, are needed.

It is important to acknowledge the limitations of our study. First, the generalizability of our findings is restricted to young male individuals with a moderate percentage of risky drinking (about twice as high compared to the general German male population of a comparable age range, Lange et al. (2017)) and no occurrence of alcohol use disorders. Despite the value of knowledge regarding healthy populations, especially for preventive purposes, the relationships found are likely to differ in populations with higher average typical alcohol use, so the presented findings need replication in samples from these populations. In addition, the male-only sample limits generalizability, and although men remain at higher risk for problematic alcohol use than women, this gender gap is narrowing, and there is a strong need for valid paradigms such as ad-libitum taste tests for laboratory studies, particularly among women (White 2020). Second, the effect of the study design including the stress induction on the findings presented cannot be excluded, although there is no conclusive evidence of moderation by the study groups. Third, the use of self-report measures of typical alcohol use and craving could be subject to recall and reporting biases (Del Boca and Darkes 2003). In this regard, it should also be noted that we used a self-administered web-based timeline follow-back to assess typical alcohol use, for which less data on validity is available compared to the interview-based timeline follow-back, although there is good evidence of high concordance between both methods in young adults (Rueger et al. 2012). Fourth, we only offered beer as the beverage containing alcohol in the ad-libitum taste test, which might not reflect the preferred drink of all participants. Moreover, these preferences could vary by drinking occasion, which could not be captured by the ad-libitum taste test design. Fifth, we have no information about the reliability of the ad-libitum taste test since there was only one measurement. This is, however, not only a limitation of this particular study but of the administration of the ad-libitum taste test in general. Finally, although the ad-libitum taste test provides a unique opportunity to study alcohol consumption behavior, it may not fully capture real-world drinking behavior. For example, participants’ behavior could be influenced by the presence of a laboratory context or by other aspects of the test environment.

Despite these limitations, our study has important implications for the application of the ad-libitum taste test in future studies. Our findings support the use of the ad-libitum taste test as a valid measure of motivation to drink alcohol in young males that is stable across several sample characteristics. However, its validity may be compromised at high levels of ad-libitum consumption. Considering these findings and the study’s limitations, future research could further refine the ad-libitum taste test as a valuable tool for assessing motivation to consume alcohol and other substances. This primarily concerns the avoidance of ceiling effects through limited amounts of offered substances as well as careful consideration of potential effects of social desirability and individual preferences for beverages containing alcohol. The potential moderating role of drinking motives, the potential involvement of impulsivity, and gender might be further targets of research focusing on the transition from alcohol craving to the initiation of alcohol use. Ultimately, these lines of research could contribute to improved laboratory studies of alcohol use behaviors and enhance our understanding of their underlying mechanisms.

## Data Availability

Datasets will be available from the corresponding author upon reasonable request.

## References

[CR1] Adams ZW, Kaiser AJ, Lynam DR (2012). Drinking motives as mediators of the impulsivity-substance use relation: pathways for negative urgency, lack of premeditation, and sensation seeking. Addict Behav.

[CR2] Bacon AK, Thomas SE (2013). Stress reactivity, social anxiety, and alcohol consumption in people with alcoholism: a laboratory study. J Dual Diagn.

[CR3] Becker G, Cherny SS (1994). Gender-controlled measures of socially desirable responding. J Clin Psychol.

[CR4] Bitsoih J, Patock-Peckham JA, Canning JR (2023). Do coping motives and perceived impaired control mediate the indirect links from childhood trauma facets to alcohol-related problems?. Behav Sci.

[CR5] Bowley C, Faricy C, Hegarty B (2013). The effects of inhibitory control training on alcohol consumption, implicit alcohol-related cognitions and brain electrical activity. Int J Psychophysiol.

[CR6] Bresin K, Mekawi Y, Verona E (2018). The effect of laboratory manipulations of negative affect on alcohol craving and use: a meta-analysis. Psychol Addict Behav J Soc Psychol Addict Behav.

[CR7] Bujarski S, Ray LA (2016). Experimental psychopathology paradigms for alcohol use disorders: applications for translational research. Behav Res Ther.

[CR8] Christiansen P, Cole JC, Field M (2012). Ego depletion increases ad-lib alcohol consumption: investigating cognitive mediators and moderators. Exp Clin Psychopharmacol.

[CR9] Colby SM, Rohsenow DJ, Monti PM (2004). Effects of tobacco deprivation on alcohol cue reactivity and drinking among young adults. Addict Behav.

[CR10] Collins RL, Kashdan TB, Koutsky JR (2008). A self-administered timeline followback to measure variations in underage drinkers’ alcohol intake and binge drinking. Addict Behav.

[CR11] Cooper ML (1994). Motivations for alcohol use among adolescents: development and validation of a four-factor model. Psychol Assess.

[CR12] Del Boca FK, Darkes J (2003). The validity of self-reports of alcohol consumption: state of the science and challenges for research. Addict Abingdon Engl.

[CR13] Fan J, Gijbels I (1996). Local polynomial modelling and its applications.

[CR14] Field M, Eastwood B (2005). Experimental manipulation of attentional bias increases the motivation to drink alcohol. Psychopharmacology.

[CR15] Field M, Quigley M (2009). Mild stress increases attentional bias in social drinkers who drink to cope: a replication and extension. Exp Clin Psychopharmacol.

[CR16] Hautzinger M, Meyer TD (2006) Depressionsdiagnostik. In: Petermann F, Eid M (eds) Handbuch der psychologischen diagnostik. Göttingen, Hogrefe, pp 540–549

[CR17] Hinz A, Daig I, Petrowski K, Brähler E (2012). Mood in the German population: norms of the multidimensional mood questionnaire MDBF. Psychother Psychosom Med Psychol.

[CR18] Jones A, Guerrieri R, Fernie G (2011). The effects of priming restrained versus disinhibited behaviour on alcohol-seeking in social drinkers. Drug Alcohol Depend.

[CR19] Jones A, Rose AK, Cole J, Field M (2013). Effects of alcohol cues on craving and ad libitum alcohol consumption in social drinkers: the role of disinhibition. J Exp Psychopathol.

[CR20] Jones KA, Chryssanthakis A, Groom MJ (2014). Impulsivity and drinking motives predict problem behaviours relating to alcohol use in university students. Addict Behav.

[CR21] Jones A, Button E, Rose AK (2016). The ad-libitum alcohol ‘taste test’: secondary analyses of potential confounds and construct validity. Psychopharmacology.

[CR22] Lange C, Manz K, Kuntz B (2017) Alcohol consumption among adults in Germany: risky drinking levels. 10.25646/2581 10.17886/RKI-GBE-2017-044 PMC10161262 37152096

[CR23] Leeman RF, Corbin WR, Fromme K (2009). Craving predicts within session drinking behavior following placebo. Pers Individ Dif.

[CR24] Leeman RF, Corbin WR, Nogueira C (2013). A human alcohol self-administration paradigm to model individual differences in impaired control over alcohol use. Exp Clin Psychopharmacol.

[CR25] Lim SS, Vos T, Flaxman AD (2012). A comparative risk assessment of burden of disease and injury attributable to 67 risk factors and risk factor clusters in 21 regions, 1990–2010: a systematic analysis for the Global Burden of Disease Study 2010. The Lancet.

[CR26] Malehi AS, Pourmotahari F, Angali KA (2015). Statistical models for the analysis of skewed healthcare cost data: a simulation study. Health Econ Rev.

[CR27] McGrath E, Jones A, Field M (2016). Acute stress increases ad-libitum alcohol consumption in heavy drinkers, but not through impaired inhibitory control. Psychopharmacology.

[CR28] McNeill AM, Monk RL, Qureshi AW (2022). Elevated ad libitum alcohol consumption following continuous theta burst stimulation to the left-dorsolateral prefrontal cortex is partially mediated by changes in craving. Cogn Affect Behav Neurosci.

[CR29] Newson RB (2012). Sensible parameters for univariate and multivariate splines. Stata J.

[CR30] Quigley BM, Collins RL (1999). The modeling of alcohol consumption: a meta-analytic review. J Stud Alcohol.

[CR31] Raabe A, Grüsser SM, Wessa M (2005). The assessment of craving: psychometric properties, factor structure and a revised version of the alcohol craving questionnaire (ACQ). Addict Abingdon Engl.

[CR32] Rehm J, Baliunas D, Borges GLG (2010). The relation between different dimensions of alcohol consumption and burden of disease: an overview. Addict Abingdon Engl.

[CR33] Royall R (1986). Model robust confidence-intervals using maximum-likelihood estimators. Int Stat Rev.

[CR34] Rueger SY, Trela CJ, Palmeri M, King AC (2012). Self-administered web-based timeline followback procedure for drinking and smoking behaviors in young adults. J Stud Alcohol Drugs.

[CR35] Spielberger CD (1983) Manual for the state-trait anxiety inventory STAI (form Y). Palo Alto, CA: consulting psychologists press

[CR36] Stahre M, Naimi T, Brewer R (2006). Measuring average alcohol consumption: the impact of including binge drinks in quantity-frequency calculations. Addict Abingdon Engl.

[CR37] Thomas SE, Bacon AK, Randall PK (2011). An acute psychosocial stressor increases drinking in non-treatment-seeking alcoholics. Psychopharmacology.

[CR38] Thomas SE, Merrill JE, von Hofe J, Magid V (2014). Coping motives for drinking affect stress reactivity but not alcohol consumption in a clinical laboratory setting. J Stud Alcohol Drugs.

[CR39] Van Dyke N, Fillmore MT (2015). Operant responding for alcohol following alcohol cue exposure in social drinkers. Addict Behav.

[CR40] White AM (2020). Gender differences in the epidemiology of alcohol use in the United States. Alcohol Res: Curr Rev.

[CR41] Wiers CE, Shumay E, Volkow ND (2015). Effects of depressive symptoms and peripheral DAT methylation on neural reactivity to alcohol cues in alcoholism. Transl Psychiatry.

[CR42] Wittgens C, Muehlhan M, Kräplin A (2022). Underlying mechanisms in the relationship between stress and alcohol consumption in regular and risky drinkers (MESA): methods and design of a randomized laboratory study. BMC Psychol.

